# Metabolomics Analysis of L-Arginine Induced Gastrointestinal Motility Disorder in Rats Using UPLC-MS After Magnolol Treatment

**DOI:** 10.3389/fphar.2019.00183

**Published:** 2019-03-01

**Authors:** Xiao Wang, Chen Zhang, Mingyue Zheng, Fei Gao, Jinming Zhang, Fang Liu

**Affiliations:** ^1^College of Pharmacy, Chengdu University of Traditional Chinese Medicine, Chengdu, China; ^2^School of Chinese Medicine, Li Ka Shing Faculty of Medicine, The University of Hong Kong, Hong Kong, China

**Keywords:** magnolol, gastrointestinal motility disorders, metabolomics, L-arginine, serotonin

## Abstract

**Background and Purpose:** Magnolol, as the main active ingredient of Traditional Chinese Medicine, can significantly improve gastrointestinal motility disorders (GMD). In the present study, metabolomics was used to investigate the mechanism of magnolol improving L-arginine induced GMD in rats.

**Experimental Approach:** SD rats were randomly divided into control group, model group and magnolol treated group. L-arginine was injected intraperitoneally in model and magnolol groups to induce GMD model. All intervention regimens were administered by oral gavage, once a day for five consecutive days. Relative gastric emptying rate and propulsive intestinal rate were measured. Metabolites in serum were analyzed based on UPLC-MS metabolomics technique.

**Results:** Magnolol significantly promoted gastric emptying and small intestinal propulsion. Compared with the model group, the level of serotonin and L-tryptophan significantly reversed (*P* < 0.05) and 22 metabolites reversed in the magnolol group. According to MetPA database analysis, magnolol has mainly affected 10 major metabolic pathways which were related to each other, Tryptophan metabolism is the most critical metabolic pathway associated with gastrointestinal tract.

**Conclusion:** These findings suggest that magnolol has a significantly promoting effect on L-arginine induced gastrointestinal motility disorder in rats, the mechanism is to reduce the production of nitric oxide to weaken the function of nitric oxide relaxing the gastrointestinal smooth muscle and increase the content of serotonin to promote gastrointestinal peristalsis and motility, secretion, absorption of nutrients.

## Introduction

Gastrointestinal motility disorders (GMD) are a group of diseases, characterized by non-specific symptoms including nausea, vomiting, bloating, abdominal discomfort or pain, constipation or diarrhea. It includes gastrointestinal dysfunction, screw weakening, and delays gastric emptying and intestinal propulsion (Beyder and Farrugia, 2012; Valentin et al., 2015). Moreover, GMD mainly consists of irritable bowel syndrome and other functional gastrointestinal disorders (Manabe et al., 2010), has been a common public health issue worldwide. Normal peristalsis and homeostatic sensory and motor mechanisms along the gastrointestinal tract result from a series of control mechanisms involving extrinsic parasympathetic and sympathetic pathways, intrinsic nervous system and the electrical and contractile properties of the smooth muscle cells (Valentin et al., 2015). Abnormalities in either pathway can cause GMD. The most commonly used effective drugs for the treatment of GMD are cisapride and domperidone. However, both of them were associated with adverse cardiovascular events (Chen et al., 2010; Arana et al., 2015).

*Magnolia Officinalis* Rehder and EH Wilson (*M. offcinalis*) are traditional Chinese medicines widely used for gastrointestinal tract motility disorder in Asian countries (Kim et al., 2017). Magnolol (5,5′-diallyl-2,2′-dihydroxybipheny) is a lignan exacted from the bark of *M. officinalis*, the compounds of which demonstrating anti-cancer, anti-stress, anti-anxiety, anti-depressant, anti-oxidant and anti-inflammatory effects (Lee et al., 2011; Zhao et al., 2017). Several studies showed that magnolol could improve the gastric emptying of a semi-solid meal and intestinal propulsive activity in mice (Wei-Wei et al., 2005). It also can prevent sepsis-induced suppression of intestinal motility in rats by modulating self-amplified inflammatory events and block oxidative stress in the intestine (Yang et al., 2008). It was also found that it can prevent lipopolysaccharide-induced septic dysmotility in mice by regulating stem cell factor (SCF)/c-kit and nitric oxide (NO) signaling to maintain functional interstitial cells of Cajal (ICCs) (Miao et al., 2013). To some extent, these reports indicate that magnolol can regulate gastrointestinal motility disorder. However, its metabolism *in vivo* is unclear, and its regulatory mechanism cannot be further explained.

Metabolomics is the science that studies the metabolic content of a sample with the aim to associate the metabolite concentrations found in the physiology of the organism or the properties of the product (Gika and Theodoridis, 2011). It adopted metabolic profiling methods for the comprehensive analysis of biological fluids, and has a great impact on the investigation of discovering biomarkers, and identifying perturbed pathways due to disease or drug treatment. Recently, some reports have described metabolomic studies using various techniques, among the analytical techniques in metabonomic research, UPLC-MS has demonstrated its great potential essentially due to the high reproducibility of measurements and sensitivity of analysis (Ramirez et al., 2013; Lee et al., 2015; Xu et al., 2016). In recent years, metabolomics technology has also contributed to the study of the mechanism of drug action. For example, Metabolomics study showed that the anti-osteoporosis effect of Achyranthes bidentata polysaccharide in ovariectomized rats was related to lipid metabolism (Zhang M. et al., 2018); It is also found that Ilex pubescens extract can prevent blood stasis in rats through arachidonic acid metabolism and glycerol phospholipid metabolism by metabolomics (Zhang Y. et al., 2018). Therefore, this study aimed to study metabolomics platform based on UPLC-MS technique to investigate the effects and mechanisms of magnolol on GMD in rats.

## Materials and Methods

### Chemicals and Reagents

Methanol, was purchased from Ourchem (Shanghai, China). Acetonitrile, was purchased from Merck (Darmstadt, Germany). Formic acid, was purchased from TCI (Shanghai, China). Double distilled water was obtained from Arium^®^ mini. Magnolol (Purity > 98%) was purchased from Chengdu Push Bio-technology Co., Ltd. (Sichuan, China). L-arginine was purchased from Biotal (United Kingdom). Dextran Blue 2000 (DB-2000) was purchased from Sigma-Aldrich (United States). Sodium Chloride Injection was obtained from Sichuan Kelun Pharmaceutical Co., Ltd. (Sichuan, China). Rat NOS2/iNOS(Nitric Oxide Synthase 2, Inducible) ELISA Kit was purchased from Elabscience Biotechnology Co., Ltd. (Wuhan, China). NO (Nitric Oxide) Assay Kit was purchased from Beyotime Biotechnology (Shanghai, China).

### Animals

Male Sprague-Dawley rats weighing 180–200 g (Chengdu Dossy Biological Technology Co., Ltd.) were under the conditions of room temperature (20–23°C), humidity (50 ± 10%), light (12 h light/dark cycle) and were free access to diet. They acclimatized for 5 days before to the start of the experiment. All experiments and procedures performed according to the Regulations of Experimental Animal Administration issued by the State Committee of Science and Technology of China and the protocol (2014DL-023) approved by the Committee on the Ethics of Animal Experiments of Chengdu University of Traditional Chinese Medicine.

### Animal Experiments and Sample Collection

After acclimatization, GMD model was induced by injected intraperitoneally with L-arginine at a dose of 5.2 g/kg on the first day and 2.6 g/kg on the second to the fifth day (Aiwu et al., 2012). Saline was injected intraperitoneally to rats in the control group for the same period. All the rats with comparable disease index were then randomly divided into two groups (*n* = 8 per group): model group: intragastric administrated with saline; magnolol group: intragastric administrated with magnolol (40 mg/kg). The rats were intragastric administrated with saline in control group. All the rats were administrated via gastric irrigation once daily for 5 days, then prohibited any food for 12 h before the experiments, but were allowed access to water freely.

On the fifth day, each group was administration 2% DB-2000 (0.4 mL) at the same time. After 30 min, the blood was collected from femoral artery. After clotting at 4°C for 2 h, the blood centrifuged at 3500 rpm for 10 min. The supernatant samples were transferred to Eppendorf tubes and immediately stored at -80°C until analysis (Tien et al., 2016).

### Gastric Emptying and Small Intestinal Transit Experiments

After the rats were bled, pylorus and cardia ligated, the stomach was taken out, the greater curvature of the stomach was cut off. The gastric residual pigment fully washed in 4 ml deionized water, centrifuged at 3500 rpm for 15 min, taken the supernatant filtrate and measure the absorbance at 620 nm with a UV756CRT spectrophotometer, the percentage of the absorbance and the mean value of the blank group is the relative gastric retention rate. Meanwhile, the distance between the pyloric sphincter to the most front of the pigments and the cecum was measured, and the ratio of the two was used as small intestinal propulsive rates.

$$\mathrm{Relative}\;\mathrm{gastric}\;\mathrm{retention}\;\mathrm{rate}\%=\frac{A_{n}}{A_{0}}\times 100\%$$

Where *A_n_* is absorbance of DB-2000 recovered from the stomach of rat, *A*_0_ is mean absorbance of recovered from the stomachs of control rats.

$$\mathrm{Intestinal}\;\mathrm{propulsive}\;\mathrm{rate}\;\left(\%\right)=\frac{\mathrm{DB}-2000\;\mathrm{advance}\;\mathrm{distance}}{\mathrm{total}\;\mathrm{length}\;\mathrm{of}\;\mathrm{the}\;\mathrm{small}\;\mathrm{intestine}}\times 100\%$$

### Metabolic Analysis of Serum Samples

#### Sample Preparation

Prior to analysis, serum samples were thawed at 4°C, transferred 100 μL of each sample into 1.5 mL centrifuge tubes, add 400 μL of methanol (precooled at -20°C) to each tube and vortex for 60 s, centrifuged for 10 min at 12000 rpm 4°C and transferred all supernatant in each tube into another 1.5 mL centrifuge tube, filtered through 0.22 μm membrane and obtain the prepared sample extracts for UPLC-MS. For the quality control (QC) samples, taken 20 μL from each prepare sample extract and mix (These QC samples were used to monitor deviations of the analytical results from these pool mixtures and compare them to the errors caused by the analytical instrument itself), used the rest of the samples for UPLC-MS test (Zelena et al., 2009; Dunn et al., 2011).

#### Analysis of Serum Samples

Chromatographic separation was accomplished in an Acquity UPLC system equipped with an ACQUITY UPLC^®^ BEH C_18_ (100 × 2.1 mm, 1.7 μm, Waters) column maintained at 40°C. The temperature of the autosampler was 4°C. Gradient elution of analytes was carried out with 0.1% formic acid in water (A) and 0.1% formic acid in acetonitrile (B) at a flow rate of 0.25 mL/min. Injection of 10 μL of each sample was done after equilibration. An increasing linear gradient of solvent B (v/v) was used as follows: 0∼1 min, 2% B; 1∼9.5 min, 2∼50% B; 9.5∼14 min, 50∼98% B; 14∼15 min, 98% B; 15∼15.5 min, 98∼2% B; 15.5∼17 min, 2%B (Sangster et al., 2006). The separated components were subsequently fragmented and analyzed using a mass spectrometer.

The ESI-MS^n^ experiments were executed on the Thermo LTQ Orbitrap XL mass spectrometer with the spray voltage of 4.8 and -4.5 kV in positive and negative modes, respectively. Sheath gas and auxiliary gas were set at 45 and 15 arbitrary units, respectively. The capillary temperature was 325°C. The voltages of capillary and tube were 35 and 50, -15 and -50 V in positive and negative modes, respectively. The Orbitrap analyzer scanned over a mass range of m/z 89∼1000 for a full scan at a mass resolution of 60000. Data-dependent acquisition MS/MS experiments were performed with CID scan. The normalized collision energy was 30 eV. Dynamic exclusion was implemented with a repeat count of 2, and exclusion duration of 15 s (Want et al., 2013).

#### Data Processing and Analysis

Converting the original data obtained by Proteowizard software (v3.0.8789) was converted into mzXML format (Smith et al., 2006). R (v3.3.2) XCMS package was used to carry out peaks identification, peaks filtration and peaks alignment (The main parameters are bw = 5, ppm = 15, and peakwidth = c (10,120), mzwid = 0.015, mzdiff = 0.01. method = “centWave”). The data matrix including information of mass to charge ratio (m/z), retention time, and intensity were obtained. In addition, the data was exported to excel for subsequent analysis. In order to allow different magnitudes of data to be compared, the intensity had been batch normalization.

According to the characteristics of metabolomics, UV (Autoscalin, mean-centering and scaled to unit variance (Thevenot et al., 2015) was used to convert the data. The multivariate statistical analysis (software SIMCA-P (v13.0) and R language ropes package) was used to analyze the data, including Principal Component Analysis (PCA), Partial Least Squares-Discriminant Analysis (PLS-DA), and Orthogonal-Partial Least Squares-Discriminant Analysis (OPLS-DA) methods.

#### Metabolite Identifications and Pathway Analysis

By screening the metabolites, the biomarkers were found. Related differential metabolite screening conditions: *p*-value ≤ 0.05+VIP ≥ 1 (Trygg, 2010), one-way ANOVA *p*-value ≤ 0.05 (Haspel et al., 2014). The identification of metabolites is first confirmed according to the exact molecular weight (molecular weight error is < 20 ppm), followed by the MS/MS fragmentation pattern to Human Metabolome Database (HMDB)^1^, Metlin^2^, massbank^3^, LipidMaps^4^, mzclound (LipidMaps) database confirm the annotation to get metabolites.

Encyclopedia of Genes and Genomes (KEGG) is a systematic analysis of gene function and genome information database (Kanehisa and Goto, 2000). MetPA is a part of metaboanalyst^5^, which is mainly based on the KEGG metabolic pathway. MetPA database can be used to analyze metabolic pathways related to metabolites.

### Detection of iNOS and NO Concentration

The concentration of iNOS was measured by ELISA using commercially available kits according to the manufacturer’s protocols. The serum level of NO was measured using a commercially available kit according to the manufacturer’s instructions.

## Results

### Effect of Magnolol Gastric Emptying and Small Intestinal Propulsion

**FIGURE 1:**
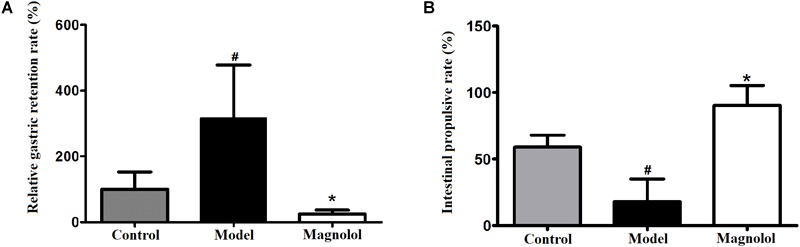
Effects on gastric emptying and intestinal propulsion. **(A)** Relative gastric remnant rate. **(B)** Intestinal propulsive rate. #Control group compared with model group *P*<0.05, ^∗^Magnolol group compared with model group *P*<0.05.

The results of gastric emptying and small intestinal transit were shown in Figure 1. Compared with the control group, the relative gastric remnant rate had significantly increased and intestinal propulsive rate had significantly reduced in model group (*P* < 0.05). The pathogenic factors of gastrointestinal motility disorder are complex. At present, there is no objective criterion for gastrointestinal motility disorder model, but atropine and L-arginine are commonly selected drugs for this model (Ren et al., 2017; Wang et al., 2018). The results of gastric emptying and intestinal propulsion also indicate that L-arginine had inhibited gastric emptying and intestinal propulsion, which indicated that the model of gastrointestinal motility disorder was successful. Compared with model group, the relative gastric remnant rate had significantly reduced and the intestinal propulsive rate had a significant increase in magnolol group. The results showed that magnolol could promote gastric emptying and small intestinal propulsion, it can effectively improve the gastrointestinal motility disorder in rats.

### Metabolomic Analysis

#### Chromatographic Peak Separated

**FIGURE 2:**
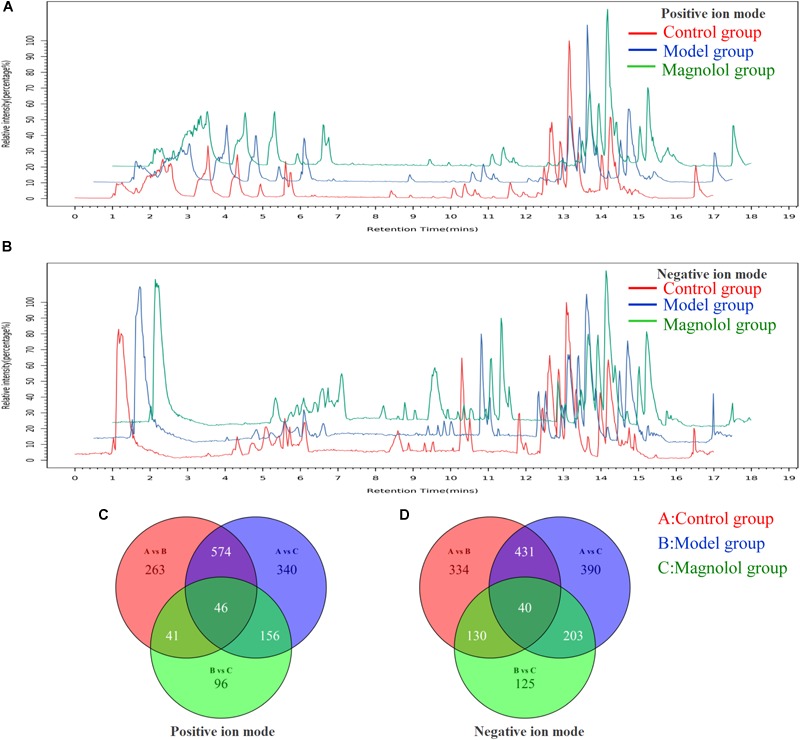
Typical sample base peak chromatogram and Venn diagrams. **(A)** Base peak chromatogram in positive ion mode. **(B)** Base peak chromatogram in negative ion mode. **(C)** Venn diagram in positive ion mode. **(D)** Venn diagram in negative ion mode.

The base peak chromatograms of typical samples in each group are shown in Figure 2A,B. The peaks of the three groups are approximately the same in both positive and negative ion modes. In total, 4535 precursor molecules were obtained in positive ion mode, and 5654 precursor molecules were obtained from negative ion mode. After analysis, the number of metabolites in each group was shown in Figure 2C,D. Under the positive ion mode, there were 924 metabolites in the control group, 1116 metabolites in the model group, and 339 metabolites in the magnolol group. And there were 603 different metabolites between the control group and the model group, 359 different metabolites between the control group and the magnolol group, 436 different metabolites between model group and the magnolol group. Under the negative ion mode, there were 935 metabolites in the control group, 1064 metabolites in the model group and 498 metabolites in magnolol group. And there were 724 different metabolites between the control group and the model group, 459 different metabolites between the control group and the magnolol group, 515 different metabolites between model group and the magnolol group. Regarding the number of differential metabolites, the difference between the control group and the magnolol group was less than that between the control group and the model group, indicated that the serum composition of the magnolol was closer to the healthy rats.

#### Quality Control and Quality Assurance

**FIGURE 3:**
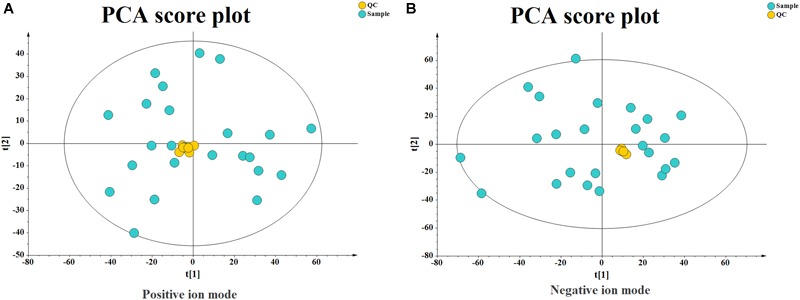
Principal Component Analysis (PCA) Score plot of quality control (QC) Samples. **(A)** At positive ion mode. **(B)** At negative ion mode.

**FIGURE 4:**
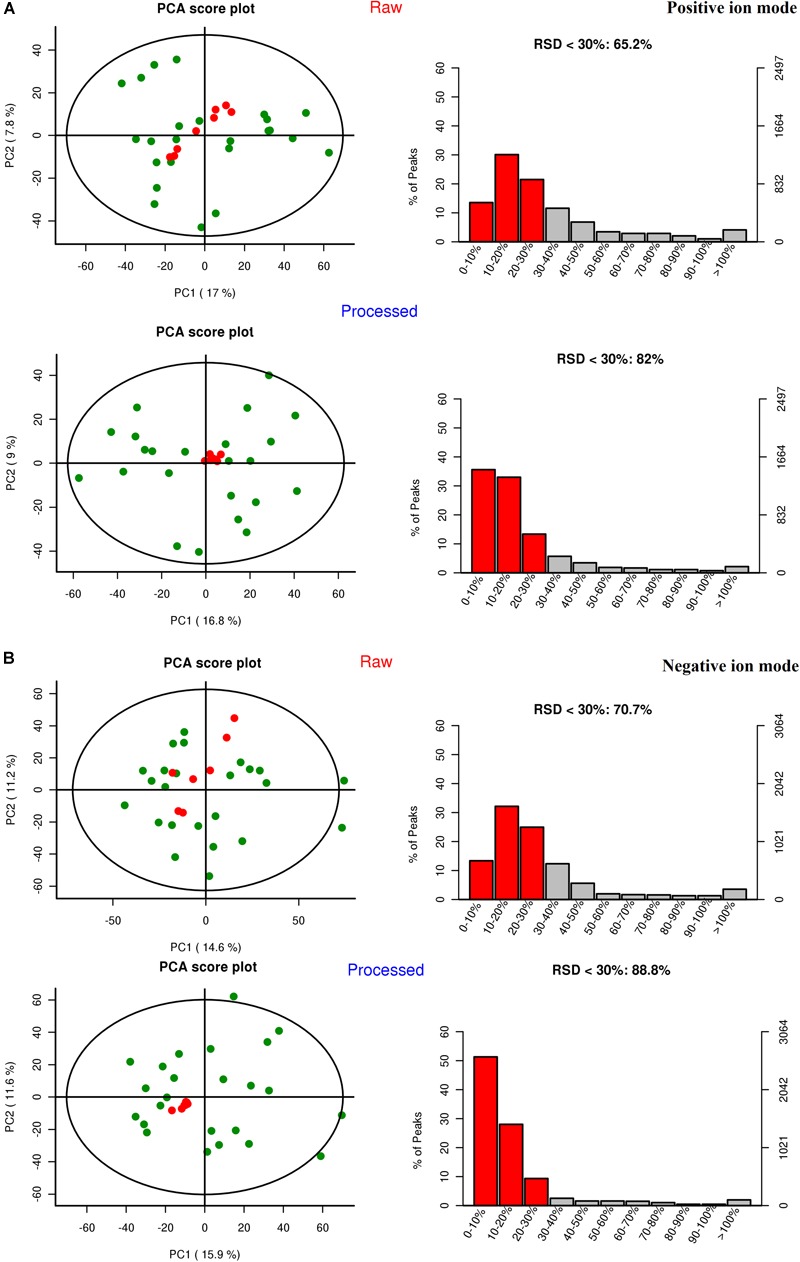
The results of the quality assurance (QA). **(A)** At positive ion mode. **(B)** At negative ion mode.

Quality control (QC) can obtain whether the system error of the whole experiment is within the controllable range. Quality assurance (QA) can eliminate unreliable variables and control analysis errors within the scope of not affecting the results of multivariate statistical analysis. Generally, if the error of QC is less than 2SD, the QC sample aggregation system is reliable. For QA, it is acceptable that RSD (%) less than 30% is recommended for biomarker analysis. Therefore, variables with RSD (%) more than 30% are eliminated in the subsequent biomarker discovery process, and thus the RSD (%) LC-MS of QA is controlled within 30% (Dunn et al., 2011). As shown in Figure 3, the QC samples are clustered relative to the experimental samples, and the QC error is less than 2 times SD. The system error in the positive ion mode is smaller than that in the negative ion mode. After quality assurance, as shown in Figure 4, At positive ion mode, RSD is less than 30% of the variables, accounting for 75.24% of the total variables; at negative ion mode, RSD is less than 30% of the variables, accounting for 80.19% of the total variables, The total proportion is more than 70% proves to be a good data set, so the experimental data are reliable.

#### Multivariate Statistical Analysis

**FIGURE 5:**
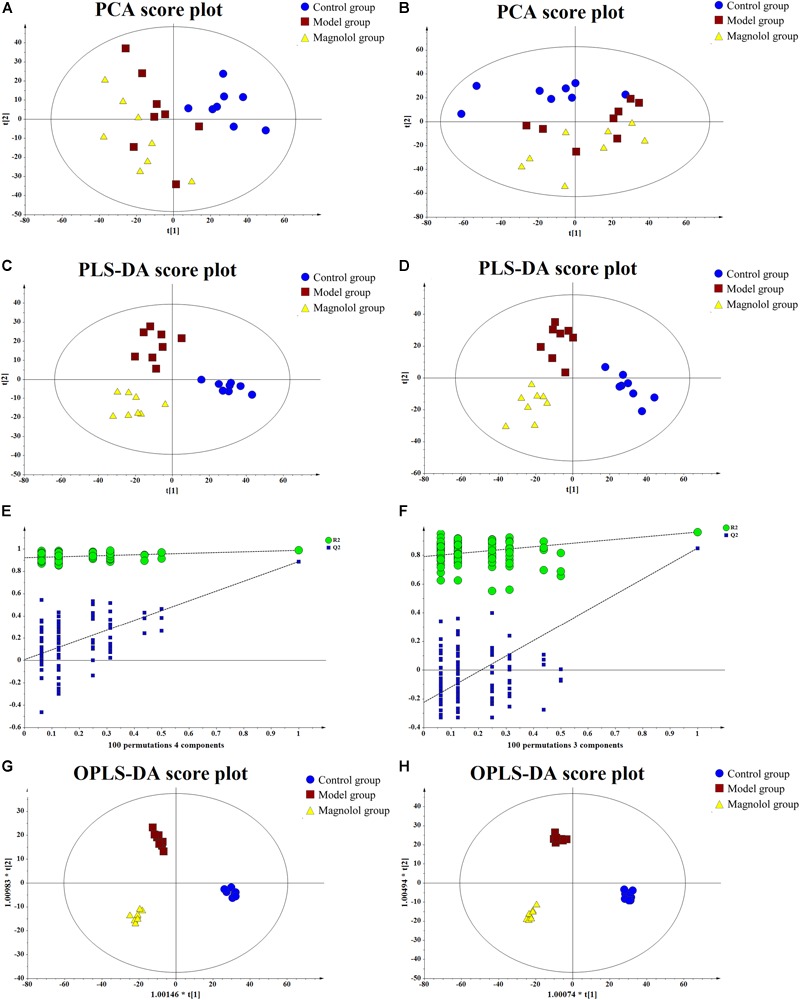
The results of multivariate statistical analysis. **(A)** The PCA score plot at positive ion mode. **(B)** The PCA score plot at negative ion mode. **(C)** The PLS-DA score plot at positive ion mode. **(D)** PLS-DA score plot at negative ion mode. **(E)** Permutations plot at positive ion mode. **(F)** Permutations plot at negative ion mode. **(G)** The OPLS-DA score plot at positive ion mode. **(H)** The OPLS-DA score plot at negative ion mode.

Good separation among the three groups was obtained using multivariate statistical analysis. PCA score plot showed the obvious separation trend between control and model groups (Figure 5A,B), which indicated that the model of gastrointestinal motility disorder was successfully established (ESI+: R2X = 0.423, Q2 = 0.0254; ESI-: R2X = 0.456, Q2 = 0.0966). To find the different compounds between groups, the PLS-DA is used to extract the information of variation between groups (Figure 5C,D). PLS-DA score plot showed that the classification effect was significant that each group separated from each other (ESI+: R2X = 0.363, R2Y = 0.986, Q2 = 0.659; ESI-: R2X = 0.343, R2Y = 0.922, Q2 = 0.650). PLS-DA not only tests the quality of the model by cross-validation, but also uses permutation test to evaluate whether the model is over-fitting (Figure 5E,F). In the positive ion mode, R2 = (0.0, 0.921), Q2 = (0.0, 0.00762); In negative ion mode, R2 = (0.0, 0.791), Q2 = (0.0, -0.225). In either mode, all blue Q2 points are lower from left to right than the original blue Q2 points, indicating that PLS-DA model has not been over-fitted. OPLS-DA was used to reduce the complexity of the model and enhance the explanatory power of the model (Figure 5G,H). The results illustrated that the metabolites in each group had been completely separated either the positive ion mode or the negative ion mode, and their VIP value was also one of the important conditions to identify the differential metabolites (ESI+: R2X = 0.365, R2Y = 0.983, Q2 = 0.632; ESI-: R2X = 0.457, R2Y = 0.989, Q2 = 0.659).

#### Screening and Identification of 30 Differential Metabolites

Potential biomarkers were screened according to *p*-value < 0.05 and VIP > 1. *p*-value was derived from Student’s *t*-test of metabolites. VIP was projected as the importance value of OPLS-DA first principal component variable. In total, 30 metabolic features majorly contributing to serum phenotype of model rats were identified (Table 1), and the majority of these metabolites were reversed by magnolol. Compared with the control group, the level of 6 metabolites significantly increased in the model group, including L-arginine, catechol, adenosine, N,N-dimethylglycine, L-valine, 4-hydroxy-L-proline, D-galactopyranose. Additionally, 24 metabolites significantly decreased. Compared with the model group, the level of 2 metabolites (serotonin and L-tryptophan) significantly reversed (*P* < 0.05) and 22 metabolites reversed in the magnolol group (Table 1).

**Table 1:** Metabolites identified in the serum of rats induced by L-Arg.

No.	Metabolite	m/z	Rt	Formula	Precursor type	Change trend	
						Model group vs. control group	Magnolol group vs. model group
1	L-arginine	175.1184	75.09590	C6H14N4O2	[M+H]+	↑#	↓
2	(R)-3-hydroxybutyrate	105.0542	110.63200	C4H8O3	[M+H]+	↓#	↓
3	citric acid	191.0193	105.26400	C6H8O7	[M-H]-	↓#	↓
4	L-glutamine	147.0757	76.14410	C5H10N2O3	[M+H]+	↓#	↑
5	Melanin	316.9463	492.77650	C18H10N2O4	[M-H]-	↓#	↑
6	suberic acid	173.0816	356.16600	C8H14O4	[M-H]-	↓#	↑
7	L-leucine	132.1014	596.03900	C6H13NO2	[M+H]+	↓#	↓
8	D-tryptophan	205.0964	257.71400	C11H12N2O2	[M+H]+	↓#	↑
9	Deethylatrazine	188.0699	257.81400	C6H10ClN5	[M+H]+	↓#	↑
10	Serotonin	177.1018	185.64200	C10H12N2O	[M+H]+	↓#	↑^∗^
11	Catechol	111.0197	781.85600	C6H6O2	[M+H]+	↑#	↑
12	L-lysine	147.1122	60.53205	C6H14N2O2	[M+H]+	↓#	↑
13	glycochenodeoxycholic acid	450.3196	556.29300	C26H43NO5	[M+H]+	↓#	↑
14	sebacic acid	201.1125	461.29800	C10H18O4	[M-H]-	↓#	↑
15	creatine zwitterions	132.0758	72.99950	C4H9N3O2	[M+H]+	↓#	↑
16	Linoleate	279.2315	894.79300	C18H32O2	[M-H]-	↓#	↑
17	Adenosine	265.9492	694.16050	C10H13N5O4	[M-H]-	↑#	↑
18	N,N-dimethylglycine	104.0702	781.00250	C4H9NO2	[M+H]+	↑#	↓
19	L-valine	118.0855	594.45700	C5H11NO2	[M+H]+	↓#	↑
20	L-ascorbate	177.0540	53.60355	C6H8O6	[M+H]+	↓#	↓
21	L-tyrosine	182.0806	77.26340	C9H11NO3	[M+H]+	↓#	↑
22	pimelic acid	159.0659	300.98100	C7H12O4	[M-H]-	↓#	↑
23	(R)-pantothenic acid	220.1169	210.10050	C9H17NO5	[M+H]+	↓#	↑
24	L-tryptophan	203.0818	258.79550	C11H12N2O2	[M-H]-	↓#	↑^∗^
25	trans-4-hydroxy-L-proline	129.9760	54.81970	C5H9NO3	[M-H]-	↓#	↑
26	glycocholic acid	466.3145	556.23100	C26H43NO6	[M+H]+	↓#	↑
27	sphingosine 1-phosphate	380.2546	718.46600	C18H38NO5P	[M+H]+	↓#	↑
28	L-pipecolic acid	129.1268	53.76355	C6H11NO2	[M+H]+	↓#	↓
29	4-hydroxy-L-proline	129.9763	274.11600	C5H9NO3	[M-H]-	↑#	↓
30	D-galactopyranose	179.0558	187.66900	C6H12O6	[M-H]-	↑#	↓

*↑: the compound is up-regulated. ↓: the compound is down-regulated. # Represents the changes in the model group compared with control group, *P* < 0.05, ^∗^ represents the changes in the Magnolol group compared with model group, *P* < 0.05.*

**FIGURE 6:**
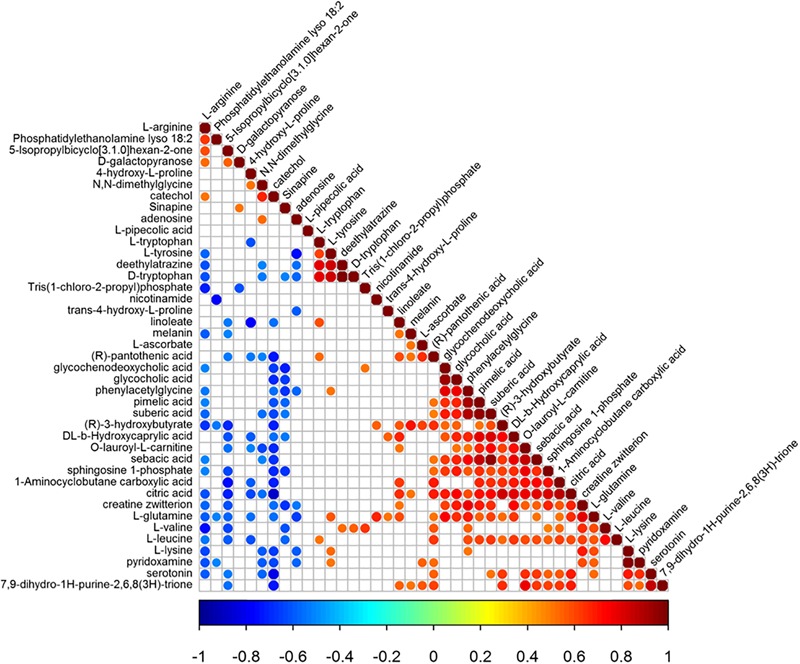
The results of metabolite-metabolite correlation analysis (Positive correlations are shown in red; negative correlations are shown in blue. The color-coded part is *p*-value < 0.05).

Pearson correlation coefficient analysis was used to analyze the metabolite-metabolite correlation among identified metabolites in control group and model group. The consistency of metabolite and metabolite trends was examined to analyze the correlation among metabolites. Its calculation method is the core function in R(v3.1.3). Meanwhile, metabolite correlation analysis was performed for significant statistical tests. The statistical test method was the cor.test function in the R language package, and a false positive check was performed on the *p*-value. Using the FDR *p*-value ≤ 0.05 was a significant correlation (Rao et al., 2016). Metabolite-metabolite correlations between the serum of control group and model group showed in Figure 6. There were 326 significant correlations, of which 228 were positively correlated and 98 were negatively correlated. Notably, amino acids dominated the significant metabolite correlations, followed by organic acids. With the intake of L-arginine, the levels of multiple metabolites in the model group changed significantly. After the administration of magnolol, the reversal of serotonin and L-tryptophan was the most obvious, and L-arginine was found to be negatively correlated with serotonin. L-tryptophan is the precursor compound of serotonin. Therefore, L-arginine is indirectly associated with L-tryptophan. Through metabolite correlation analysis, each individual metabolite is interrelated and can know the changes of the quantity of each other.

#### Metabolic Pathway Analysis

**FIGURE 7:**
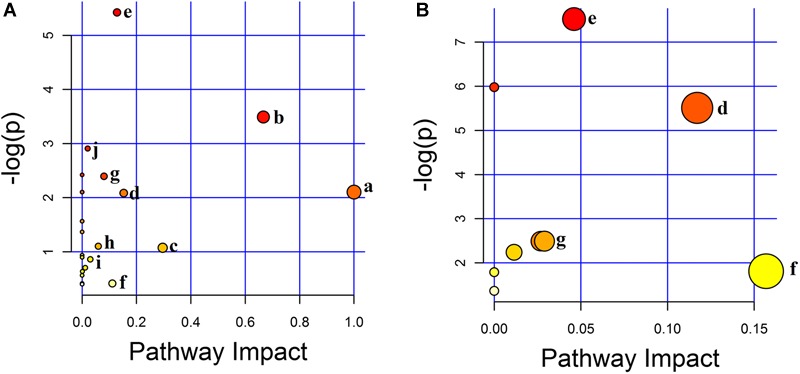
The result of metabolic pathway analysis. **(A)** The main metabolic pathways in the three groups. **(B)** The main metabolic pathways affected by magnolol (a–j represents the metabolic pathway details shown in Table 2).

**FIGURE 8:**
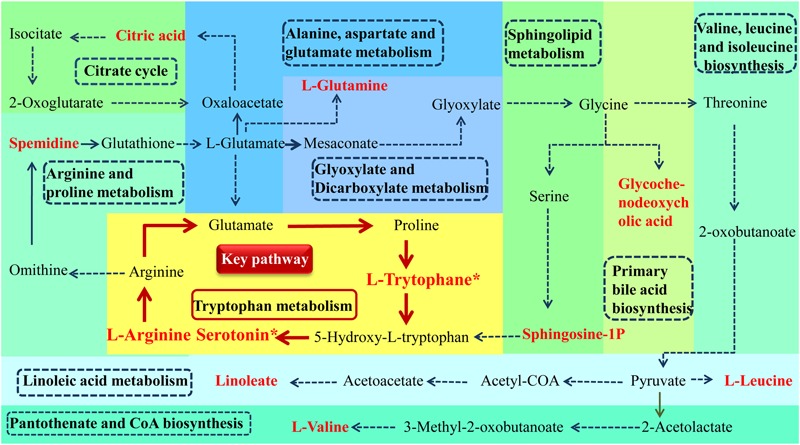
Metabolism pathway networks. (Different color plates represent different metabolic pathways. the name of pathway is in the dotted line frame. Metabolites name with red bold represent the differential metabolites which was identified (^∗^*P* < 0.05, magnolol group compared with model group).

According to MetPA database analysis, there were 10 main metabolic pathways in the three groups, which were Linoleic acid metabolism; Valine, leucine and isoleucine biosynthesis; Glyoxylate and dicarboxylate metabolism; Alanine, aspartate and glutamate metabolism; Arginine and proline metabolism; Tryptophan metabolism; Citrate cycle (TCA cycle); Primary bile acid biosynthesis; Sphingolipid metabolism; Pantothenate and CoA biosynthesis (Figure 7A). Magnolol mainly affects four of these pathways, namely Tryptophan metabolism, Alanine, aspartate and glutamate metabolism, Arginine and proline metabolism and Citrate cycle (TCA cycle), among them, tryptophan metabolic pathway has the greatest impact. (Figure 7B). The enrichment analysis of KEGG metabolic pathway result was shown in Table 2. By consulting the KEGG database, the metabolic pathways were linked through the differential metabolites to form a metabolic pathway network (Figure 8). It can be seen that magnolol not only affected a single metabolic pathway but also formed a complex network of metabolic pathways through some interrelated metabolic pathways to achieve the purpose of treating GMD. Tryptophan metabolism is the core pathway to achieve a therapeutic effect, and other metabolic pathways play a supplementary role. Magnolol significantly affected L-tryptophan and serotonin. These two substances were both in the tryptophan metabolism pathway, and tryptophan metabolism pathway was directly related to gastrointestinal motility. From the metabolism pathway network, it could be seen that L-arginine has connected with tryptophan pathway through arginine and proline metabolism and alanine, aspartate and glutamate metabolism, and ultimately affects serotonin production. The content of L-arginine in the serum of model group increased, serotonin production was inhibited, magnolol significantly increased serotonin level, and made gastrointestinal tract movement normal in rats.

**Table 2:** Enrichment analysis of Encyclopedia of Genes and Genomes (KEGG) metabolic pathway.

No.	Metabolic Pathway	Total	Raw p	-log(*p*)	Impact in three groups	Impact in magnolol	No. in Figure 7
1	Arginine and proline metabolism	44	0.004409	5.424	0.12831	0.04603	e
2	Valine, leucine and isoleucine biosynthesis	11	0.030489	3.49	0.66666	–	b
3	Pantothenate and CoA biosynthesis	15	0.054587	2.908	0.02041	–	j
4	Citrate cycle (TCA cycle)	20	0.091216	2.395	0.08044	0.02688	g
5	Linoleic acid metabolism	5	0.12213	2.103	1	–	a
6	Alanine, aspartate and glutamate metabolism	24	0.12439	2.084	0.15295	0.11708	d
7	Primary bile acid biosynthesis	46	0.33233	1.102	0.05952	–	h
8	Glyoxylate and dicarboxylate metabolism	16	0.34195	1.073	0.2963	–	c
9	Sphingolipid metabolism	21	0.4232	0.86	0.03008	–	i
10	Tryptophan metabolism	41	0.66117	0.414	0.11122	0.15684	f

### Magnolol Decreases iNOS Expression and Suppresses NO Content in GMD Rats

**FIGURE 9:**
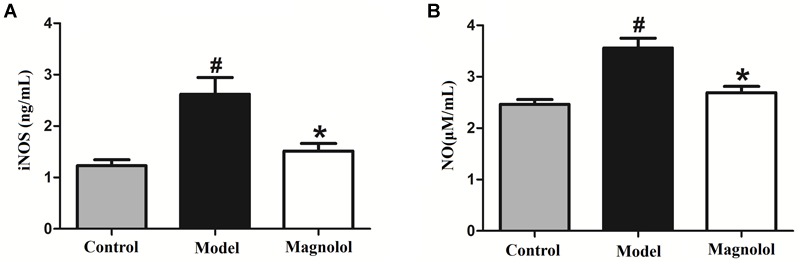
Magnolol prevents the L-arginine induced increase in iNOS and NO. **(A)** Concentrations of NO in serum in three groups. **(B)** Concentrations of iNOS in serum in three groups. #Control group compared with model group *P*<0.05, ^∗^Magnolol group compared with model group *P*<0.05.

We examined whether magnolol treatment affects the expression of iNOS using ELISA (Figure 9A). iNOS expression was significantly upregulated in the model group compared with the control group. Magnolol significantly suppressed the expression of iNOS in GMD rats. The slight increase in iNOS expression seen in magnolol group compared to control group was not statistically significant.

We also tested whether magnolol affects the NO content in the serum of GMD rats (Figure 9B). NO concentration was significantly increased in L-arginine treated animals compared with control animals. Magnolol significantly reduced the increase in NO content in GMD rats. The slight increase in NO content seen in magnolol group compared to control group was not statistically significant. The results showed that magnolol has prevented the L-arginine induced increase in iNOS and NO.

## Discussion

Magnolol has a good therapeutic effect on L-arginine induced gastrointestinal motility disorder in rats, which is supported by the following findings. (1)magnolol promoted gastric emptying; (2)magnolol promoted small intestinal propulsion; (3)magnolol significantly increased the level of serotonin and L-tryptophan in GMD rats; and (4) magnolol affected multiple metabolic pathways associated with the gastrointestinal tract *in vivo*, and tryptophan metabolism pathway played a major role in treatment. A recent study has shown that magnolol effectively improved the symptoms of Dextran Sulfate Sodium-Induced colitis in mice and its underlying mechanisms are associated with the restoring of tryptophan metabolites that inhibit the colonic inflammation (Zhao et al., 2017). It also found that magnolol acted on the tryptophan metabolism pathway, but it focused on the analysis of the kynurenine synthesis associated with anti-inflammatory effects and did not find the importance of serotonin for the gastrointestinal tract. Thus, this study used the metabolomics to study the mechanism of magnolol in the treatment of L-arginine induced GMD rats. According to the author’s knowledge, this study was the first time that a network of its metabolic pathway *in vivo* pathways was successfully constructed.

Rats were injected with L-arginine to cause GMD successfully. Yu et al. (2017) demonstrated L-arginine induced obvious functional disorders of the gastrointestinal tract, represented by significantly delayed gastric emptying and intestinal propulsion rates of mice. L-arginine is a multipurpose amino acid that also serves as a precursor for multiple metabolites, including polyamines and NO (Kim et al., 2018). L-arginine is the substrate for four enzymes, several of which exist as multiple isoforms: iNOS, arginases, glycine aminotransferase, and L-arginine decarboxylase. After arginine enters mammalian cells through the membrane-bound transporters CAT1 and CAT2B, it is metabolized by one of the iNOS to produce NO, which is a non-cholinergic and non-adrenergic neurotransmitter, can relax the smooth muscle and cause gastrointestinal motility to weaken. It plays an inhibitory role in gastrointestinal motility (Yu et al., 2017). The content of L-arginine in the model group increased significantly, magnolol reduced the amount of L-arginine in the model rats, reduced the production of NO, and alleviated the inhibition of gastrointestinal tract.

Through the metabolic pathway network, 11 red differential metabolites play an important role in linking, so that the metabolic pathways no longer exist alone, but constitute a whole. The 11 red differential metabolites including L-Arginine, Spermidine, L-Glutamine, Citric acid, Glycochenodeoxycholic acid, L-Leucine, L-Valine, Linoleate, Sphingosine-1P, L-Trytophane^∗^, Serotonin^∗^. L-arginine and Spermidine regulated gastrointestinal motility by affecting iNOS in the same metabolic pathway (Rodriguez-Gomez et al., 2016). L-arginine and L-glutamine are linked through two metabolic pathways. L-glutamine has protective effects on enteric neurons (Vicentini et al., 2016), most of the effects of L-glutamine on nerve cells are due to its conversion into glutathione which exerts an antioxidant effect through various mechanisms (Aoyama et al., 2008; Hermes-Uliana et al., 2014), comprising modulation in calcium homeostasis, apoptosis, and the intestinal immune system (Kuhn et al., 2010; Michalak et al., 2015). L-glutamine also has been shown to have antioxidant effects through influencing mechanisms of glutathione (GSH) synthesis and recycling (Chen and Herrup, 2012). The change of L-glutamine is not only related to the gastrointestinal function of magnolol, but also related to the antioxidant effect of magnolol. In addition, TCA cycle is an important energy metabolic pathway, and citric acid is one of its metabolites. Glycochenodeoxycholic acid is a metabolite of primary bile acid biosynthesis, which has a certain connection with the metabolism of bile acids. L-Leucine and L-Valine are branched-chain amino acids, have a protective effect on muscle (Maki et al., 2012).

The direct correlation of magnolol to the gastrointestinal tract is a tryptophan metabolic pathway. Sphingosine-1P, L-Trytophane^∗^ are associated with the pathway and play a role in regulating gastrointestinal motility by producing serotonin. Serotonin (5-HT), a monoamine hormone and neurotransmitter, is an essential gastrointestinal modulator whose effects regulate the intestinal physiology (Layunta et al., 2018), such as peristalsis and motility, secretion, absorption of nutrients (Mawe and Hoffman, 2013). Serotonin is synthesized in the central nervous system and the gastrointestinal tract where it is secreted from enteroendocrine cells. Its biosynthesis is regulated by two isoforms of the enzyme tryptophan hydroxylase (TPH) of which TPH1 is localized predominantly in gastrointestinal enteroendocrine cells. Serotonin activates the peristaltic reflexes, regulates gastrointestinal motility, and has a role in intestinal inflammation. Gastrointestinal 5-HT receptors are targets for the pharmacological management of gastrointestinal disorders that present with motility disturbances (Swami and Weber, 2018). The 5-HT4 receptor is expressed in various cell types in the colon and it possesses mainly prokinetic actions. 5-HT7 receptors are usually located near the 5-HT4 receptors where they help to augment their effects. Additionally, 5-HT3 receptors are localized throughout the gastrointestinal tract including enterocytes and the nerves of the myenteric plexus (Yaakob et al., 2015). Moreover, serotonin plays an important role in regulating the gastrointestinal tract by combining with various receptors. One of the mechanisms of magnolol in the treatment of gastrointestinal motility disorder is to increase serotonin content, promote gastrointestinal peristalsis and motility, secretion, absorption of nutrients and restore gastrointestinal motility to normal.

## Conclusion

Magnolol can significantly improve gastric emptying and intestinal propulsion in rats with GMD. A variety of metabolic pathways were involved to achieve this effect. Among them, arginine and proline metabolism pathway would be the key metabolic pathway to play a therapeutic effect by reducing the production of L-arginine and NO, and Tryptophan metabolism would be the key metabolic pathway to play a therapeutic effect by increasing the content of serotonin. We propose the magnolol may rely on the role of relaxing gastrointestinal smooth muscle by NO and promoting gastrointestinal peristalsis and motility by serotonin to improve L-Arginine induced GMD in rats.

## Author Contributions

XW contributed to the animal experiments, prepared and revised the manuscript. JZ and FL conceived and designed the experiments. CZ, MZ, and FG analyzed the data and wrote the manuscript.

## Conflict of Interest Statement

The authors declare that the research was conducted in the absence of any commercial or financial relationships that could be construed as a potential conflict of interest.

## Footnotes

1 http://www.hmdb.ca

2 http://metlin.scripps.edu

3 http://www.massbank.jp/

4 http://www.lipidmaps.org

5 www.metaboanalyst.ca
